# Determining the Levels of Four Phenylethanoid Glycosides and Five Triterpene Acids in Liuwei Dihuang Capsule Using Solid Phase Extraction with HPLC-UV

**DOI:** 10.1155/2019/7609438

**Published:** 2019-11-07

**Authors:** Yong Zhang, Xian-Liang Zou, Yong-Li Wang, Lu Gao, Gui-Xin Chou

**Affiliations:** ^1^Institute of Chinese Materia Medica, Shanghai University of Traditional Chinese Medicine, Shanghai, China; ^2^Shanghai R&D Center for Standardization of Chinese Medicines, Shanghai, China; ^3^Xiuzheng Pharmaceutical Group Co., Ltd., Tonghua 134001, China

## Abstract

In this study, we used quantitative high-performance liquid chromatography equipped with an ultraviolet detector (HPLC-UV) and solid phase extraction (SPE) to determine the levels of four phenylethanoid glycosides and five triterpene acids in Liuwei Dihuang capsules (LDCs). LDCs were methanol-extracted and purified using a 500 mg/6 mL silica-based C18 SPE cartridge. Two elutions were analyzed on a ChromCore C18 column under two HPLC conditions. To improve the pretreatment clean-up, an array of silica- and polymer-based SPE cartridges were compared. Both wash and elution steps were also optimized to achieve the highest removal of impurities. Under optimal chromatographic conditions, good linearity was achieved for all compounds (correlation coefficient of *r* ≥ 0.999), with a quantification limit ranging from 0.0076 to 0.418 *μ*g/mL. The method had satisfactory efficiency and reproducibility with recovery rates ranging from 91.6 to 99.3% with a relative standard deviation below 1.5%. Taken together, this demonstrated SPE as a suitable extension of HPLC-UV for the determination of phenylethanoid glycosides and triterpene acids in complex LDCs.

## 1. Introduction

Liuwei Dihuang (LD) is one of the most established traditional Chinese medicines that have been used for over a thousand years to treat backache, alopecia, menoxenia, and waist/knee pain [1]. LD consists of six crude herbs including *Radix rehmannia*, *Fructus corni*, *Rhizoma dioscoreae*, *Rhizoma alismatis*, *Cortex moutan*, and *Poria cocos* at a ratio of 8 : 4 : 4 : 3 : 3 : 3. Studies have shown that LD has therapeutic benefits for hypertension, diabetes, tuberculosis, neurosis, nephritis, neurasthenia, dementia, and Parkinson's disease [2–4].

Traditionally, LD is prepared as pills or in decoctions. Modern pharmaceutical technologies now produce LD capsules (LDCs) that permit oral administration and ease of transportation/storage. Over 100 pharmaceutical manufacturers currently produce LDCs in China. As we know, the quality of herbal medicines correlates with the levels of their chemical constituents. These can differ according to environmental conditions, resulting in variable clinical effects. According to the Chinese Pharmacopoeia 2015, the significant specification for LDC is to determine the minimum content of paeonol and ursolic acid using HPLC and HPTLC for quality control (QC) [5], only *Cortex moutan* and *Fructus corni* are assessed during LDC preparation, but these do not represent the quality, safety, and efficacy of the specific LDC product totally.

Recent studies have shown that the bioactive components of LDC include various phenylethanoid glycosides and triterpene acids which originate from herbal *Radix rehmannia* and herbal *Poria cocos*, respectively. Both constituents possess antibacterial, anti-inflammatory, and antioxidative properties and can inhibit tyrosinase activity [6–12]. As such, phenylethanoid glycosides and triterpene acids should be considered during the QC of LDC. Previous QC approaches focus solely on the bioactive markers of morofficianloside, monoterpenes, and phenolics, originated from herbal *Cortex moutan* and herbal *Fructus corni* in LD preparations. These studies used HPLC coupled to diode array detectors for the simultaneous determination of eight constituents in LD pills [13], employed micellar electrokinetic chromatography to measure bioactive constituents in LD pills [14], used HPLC-MS to detect bioactive compounds in LD pills [15], and employed HPLC-UV for the determination of specific constituents in LD preparations [16]. These methods, however, do not reflect the quality of *Radix rehmannia* and *Poria cocos* in LDC. To date, the determination of phenylethanoid glycosides and triterpene acids in LDC have not been reported due to poor sensitivity of the HPLC-UV method. It is now necessary to develop appropriate HPLC-UV-based methods that allow a consistent quality of product from different manufacturers. Recently, solid phase extraction (SPE) has emerged as an effective pretreatment technique that can enrich and purify samples for complex composition analysis [17, 18]. Thus, the combination of SPE and HPLC-UV can not only improve sensitivity but also eliminate interference during the analysis of herbal formulas.

In this study, we have performed the first quantification of four phenylethanoid glycosides and five triterpene acids in LDC samples through established SPE techniques. The methods were applied to 18 batches of LDC samples obtained from six independent manufacturers subsequently.

## 2. Experimental Procedures

### 2.1. Materials and Reagents

**Figure 1:**
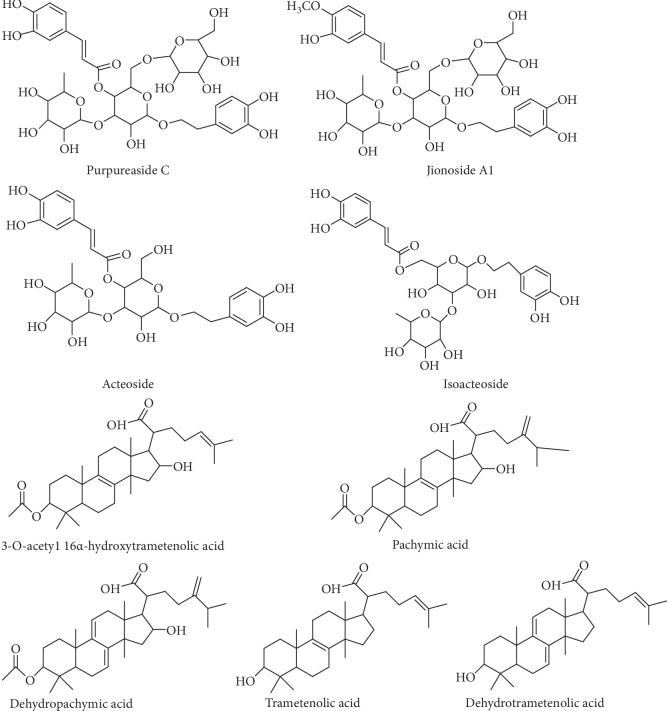
Structure of four phenylethanoid glycosides and five triterpene acids.

Standards of purpureaside C, jionoside A1, acteoside, isoacteoside, 3-O-acetyl-16*α*-hydroxytrametenolic acid, dehydropachymic acid, pachymic acid, trametenolic acid, and dehydrotrametenolic acid were obtained from the Shanghai R&D Center for the Standardization of Chinese Medicines. The purity of the chemicals was 99% or higher. The structures of four phenylethanoid glycosides and five triterpene acids are shown in Figure 1. SelectCore C18 (500 mg/6 mL), SelectCore HLB (200 mg/6 mL), Welchrom Alumina-N (500 mg/6 mL), and Welchrom PSA (500 mg/6 mL) were independently purchased from NanoChrom Technologies (Suzhou, China) and Welch Materials (Shanghai, China). They were conditioned through washing with 5 mL of methanol and 5 mL of ultrapure water prior to use. HPLC-grade methanol and acetonitrile were purchased from Fisher Scientific (Whitby, Canada). Phosphoric acid and other analytical reagents were purchased from Aladdin (Shanghai, China). Ultrapure water was generated from the Milli-Q system (Millipore, MA, USA).

### 2.2. Preparation of Standard Solutions

Standard solutions (I) were prepared in methanol with the following concentrations: 15 *μ*g/mL purpureaside C; 15 *μ*g/mL jionoside A1; 20 *μ*g/mL acteoside; and 12 *μ*g/mL isoacteoside. Standard solutions (II) were prepared in methanol with the following concentrations: 5 *μ*g/mL 3-O-acetyl 16*α*-hydroxytrametenolic acid; 20 *μ*g/mL dehydropachymic acid; 80 *μ*g/mL pachymic acid; 20 *μ*g/mL trametenolic acid; and 20 *μ*g/mL dehydrotrametenolic acid.

### 2.3. Sample Preparation

Eighteen batches of LDC (marked as samples LDC01–18) were collected from six Chinese medicinal manufacturers: T Pharmaceutical Co., Ltd. (samples LDC 01–03), X Pharmaceutical Co., Ltd. (samples LDC 04–06), Y Pharmaceutical Co., Ltd. (samples LDC 07–09), J Pharmaceutical Co., Ltd. (samples LDC10–12), R Pharmaceutical Co., Ltd. (samples LDC13–15), and P Pharmaceutical Co., Ltd. (samples LDC16–18). Negative samples (LDC lacking *Radix rehmannia* and LDC lacking *Poria cocos*) were produced in our laboratory.

### 2.4. Preparation of Sample Solutions

Fine LDC powder (1.5 g) was macerated in 50 mL of MeOH and extracted by ultrasonication for 30 min. After adjustment to its initial weight in MeOH, the mixture was centrifuged for 5 min at 6000 rpm. A total of 10 mL was then evaporated at 50°C, and the residue was dissolved in 10 mL of 10% MeOH. The mixture was loaded onto the SPE cartridge (SelectCore C18 500 mg/6 mL) at a controlled flow rate of 3 seconds per drop. The cartridge was washed with 20 mL of 10% MeOH, and the first components were eluted in 20 mL of 40% MeOH. Eluted samples were evaporated at 50°C and the residue was dissolved in 1 mL of MeOH (LDC solution A for the determination of the four phenylethanoids). The cartridge was then washed in 20 mL of 60% MeOH and the second components were eluted in 20 mL of 100% MeOH. The eluted solution was evaporated at 50°C and the residue was dissolved in 1 mL of MeOH (LDC solution B for the determination of the five triterpene acids).

The fine powder of the negative sample (without *Radix rehmannia*) (1.1 g) was macerated in 50 mL of MeOH and extracted with ultrasonicfication for 30 min. After adjustment to the initial weight in MeOH, samples were centrifuged for 5 min at 6000 rpm, 10 mL of the sample was evaporated at 50°C, and the residue was dissolved in 10 mL of 10% MeOH. The mixture was loaded onto the SPE cartridge (SelectCore C18 500 mg/6 mL). After washing the cartridge with 20 mL of 10% MeOH and 20 mL of 40% MeOH, the eluted solutions were collected and evaporated at 50°C. The residues were dissolved in 1 mL of MeOH (negative sample solution A).

The fine powder of the negative sample (without *Poria cocos*) (1.3 g) was prepared as above and loaded onto the SPE cartridge (SelectCore C18 500 mg/6 mL). The cartridge was washed in 20 mL of 10% MeOH, 20 mL of 40% MeOH, and 20 mL of 60% MeOH and eluted in 20 mL of 100% MeOH. The solution was evaporated at 50°C, and the residue was dissolved in 1 mL of MeOH (negative sample solution B).

The dried powder of *Radix Rehmanniae* and *Poria cocos* samples was pulverized and sifted through a 0.45 mm sieve. Approximately 1 g of dried powder was extracted in 50 mL of MeOH and sonicated for 30 min at 30°C. Supernatants were then combined and evaporated at 50°C. The residue was dissolved in 10 mL of methanol and used as the herbal Radix Rehmanniae and herbal *Poria cocos* test solutions.

### 2.5. Apparatus and Analytical Methods

HPLC analysis was performed on an Agilent 1260 liquid chromatography system that included a quaternary pump, thermostatic oven, thermostatic autosampler, and a UV detector. Separation was performed on ChromCore™ C18 columns (250 mm × 4.6 mm, 5 *μ*m). For the analysis of phenylethanoid glycosides, gradient program A was employed at a flow rate of 1 mL/min by combining solvent A (0.01% *v/v* phosphoric acid) and solvent B (acetonitrile) as follows: 0–40 min, 12–24% B. A pre-equilibration period of 10 min was established between individual runs. The detection wavelength was 334 nm and the column temperature was maintained at 30°C. The injection volume was 10 *μ*L.

For the analysis of triterpene acid composition, gradient program B was employed at a flow rate of 1 mL/min by combining solvent A (0.01% *v/v* phosphoric acid) and solvent B (acetonitrile) as follows: 0–25 min, 70–95% B, 25–35 min, 95% B. A pre-equilibration period of 10 min was established between individual runs. The detection wavelength was 210 nm and the column temperature was maintained at 30°C. The injection volume was 10 *μ*L.

## 3. Results and Discussion

### 3.1. Optimization of the Chromatographic Conditions

**Figure 2:**
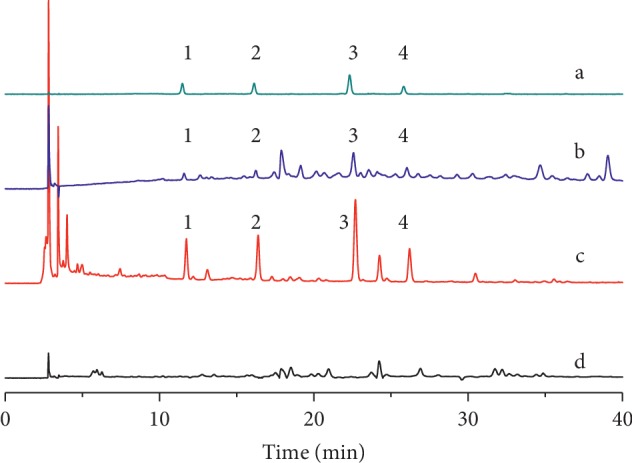
HPLC-UV chromatogram of (a) four phenylethanoid glycosides mixed standards, (b) LDC solution A, (c) herbal Radix Rehmanniae test solution, and (d) negative sample solution A with gradient program A (1) purpureaside C, (2) jionoside A1, (3) acteoside, and (4) isoacteoside.

**Figure 3:**
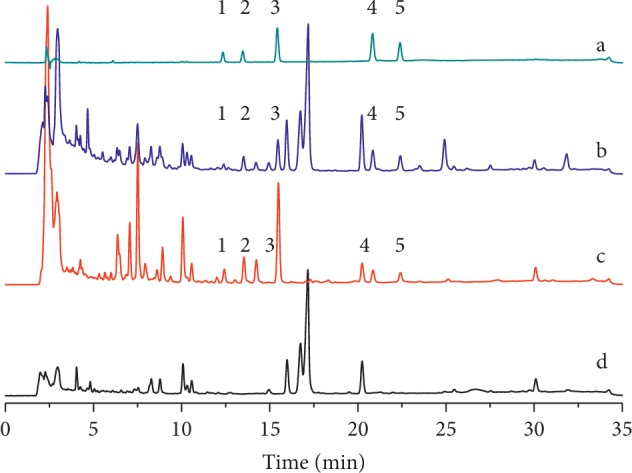
HPLC-UV chromatogram of (a) five triterpene acids mixed standards, (b) LDC solution B, (c) herbal *Poria cocos* test solution, (d) negative sample solution B with gradient program B, (1) 3-O-acetyl 16*α*-hydroxytrametenolic acid, (2) dehydropachymic acid, (3) pachymic acid, (4) trametenolic acid, and (5) dehydrotrametenolic acid.

For gradient elutions, acetonitrile and methanol were tested as organic modifiers. Acetonitrile was selected as it produces a higher signal response and lower background noise compared to MeOH. A small volume of acid was added to the mobile phase to improve the peak shape and restrain peak tailing in the LDC extracts. Aqueous phosphoric acid solutions (0%, 0.1%, and 0.2%) were then compared. The nine compounds could be separated at baseline in 0.1% phosphoric acid. To obtain optimal resolution of the C18 column and to shorten the analysis time, different gradient eluents of the mobile phase consisting of phosphoric acid and acetonitrile were studied. We tried to develop a gradient HPLC elution method to analyze nine compounds by switching the wavelength. However, due to the large differences in polarity of these nine target components, it must take more than 60 min to achieve baseline separation and accompanied with sharp fluctuations in the baseline because of wavelength switching. Thus, two gradient elution HPLC methods must be developed. In this study, gradient program A was adjusted to ensure that the four phenylethanoid glycosides from *Radix rehmannia* could achieve baseline separation in the LDC sample without negative *Radix rehmannia* interference (Figure 2). Gradient program B was adjusted to ensure that the five triterpene acids originating from herbal *Poria cocos* could attain baseline separation in the LDC sample without *Poria cocos* sample interference (Figure 3).

### 3.2. Comparison of Different Solid Phases of Phenylethanoid Glycosides

**Figure 4:**
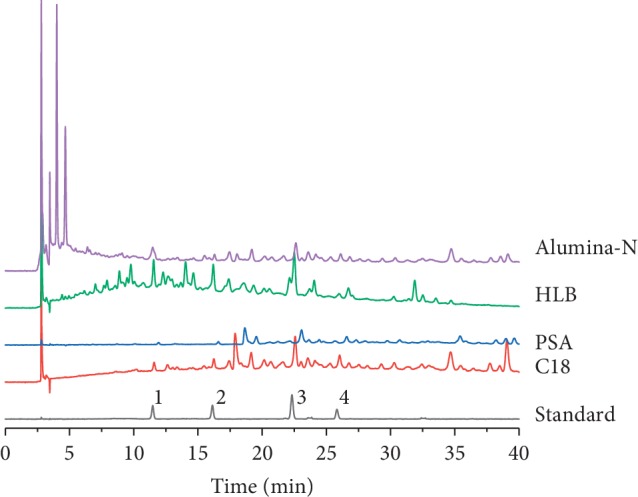
Comparison of HPLC chromatograms obtained using different SPE methods for analysis four phenylethanoid glycosides in LDC. (1) Purpureaside C, (2) jionoside A1, (3) acteoside, and (4) isoacteoside.

Due to the complexity of LDC, sample pretreatment extraction methods with high levels of clean-up, enrichment, and recovery are necessary. To evaluate the effects of different SPE cartridges on the enrichment of phenylethanoid glycosides during LDC pretreatment, four SPE cartridges, namely, silica-based C18 (SelectCore C18 500 mg/6 mL), silica-based primary secondary amine (Welchrom PSA 500 mg/6 mL), polymer-based hydrophilic lipophilic balance (SelectCore HLB 200 mg/6 mL), and neutral alumina (Welchrom Alumina-N 500 mg/6 mL) were studied. The comparative chromatograms and recovery of the target compounds are indicated in Figure 4 and Table 1. C18 and HLB SPE cartridges produced high levels of phenylethanoid glycoside recovery, which exceeded 90%. However, LDC following HLB pretreatment led to high levels of impurity and interference compared to C18. These results indicate that C18 cartridges can be used for the quantitative analyses of phenylethanoid glycosides in LDC.

### 3.3. Optimization of the SPE Pretreatment Method for Phenylethanoid Glycosides

**Figure 5:**
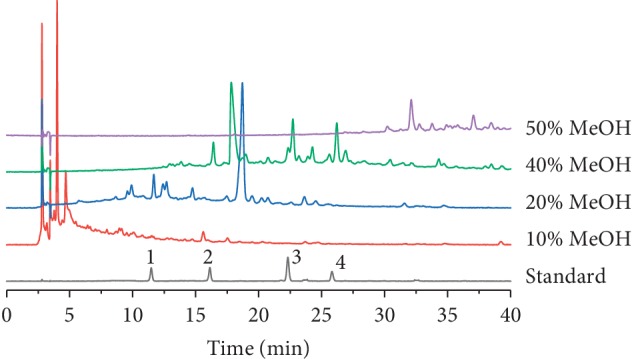
Comparison of HPLC chromatograms obtained using different compositions of methanol for analysis four phenylethanoid glycosides in LDC. (1) Purpureaside C, (2) jionoside A1, (3) acteoside, and (4) isoacteoside.

Due to the hydrophilic nature of phenylethanoid glycosides, the effects of 10%, 20%, 40%, and 50% MeOH on the enrichment efficiency of the four phenylethanoid glycosides were investigated. As shown in Figure 5, 20% MeOH led to loss of purpureaside C, while 40% MeOH produced high levels of recovery of the four phenylethanoid glycosides. No further increase was observed in 50% MeOH. From these results, 10% and 40% MeOH were selected for washing and elution steps, respectively.

### 3.4. Comparison of the Different Solid Phases of Triterpene Acids

**Figure 6:**
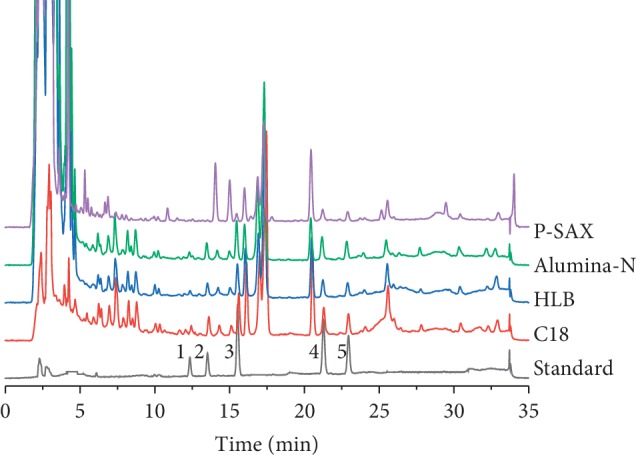
Comparison of HPLC chromatograms obtained using different SPE methods for analysis five triterpene acids in LDC. (1) 3-O-Acetyl 16*α*-hydroxytrametenolic acid, (2) dehydropachymic acid, (3) pachymic acid, (4) trametenolic acid, and (5) dehydrotrametenolic acid.

To evaluate the effects of the different SPE cartridges on the enrichment of triterpene acids during LDC pretreatment, silica-based C18 (SelectCore C18 500 mg/6 mL), polymer-based hydrophilic lipophilic balance (SelectCore HLB 200 mg/6 mL), polymer-based strong anion exchange (Welchrom P-SAX 200 mg/6 mL), and neutral alumina (Welchrom Alumina-N 500 mg/6 mL) were assessed. The chromatograms and target compound recovery are shown in Figure 6 and Table 1. The C18 and HLB SPE cartridges produced over 90% recovery of the five triterpene acids. Considering the convenience and low costs of the SPE process, C18 cartridges were chosen for the quantitative analyses of triterpene acids in LDC.

### 3.5. Optimization of the SPE Methods for Triterpene Acids

**Figure 7:**
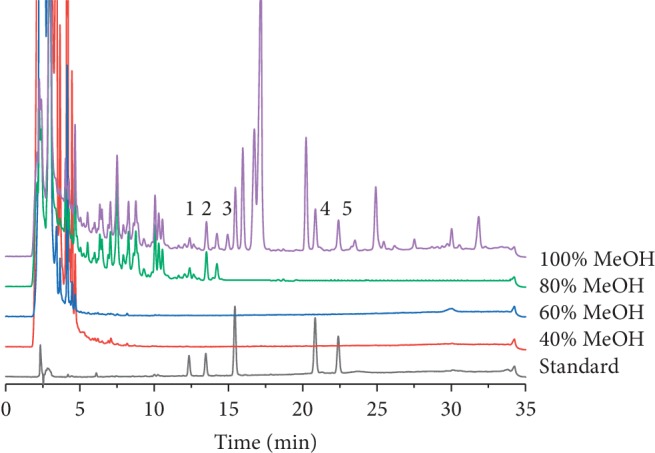
Comparison of HPLC chromatograms obtained using different composition of methanol for analysis five triterpene acids in LDC. (1) 3-O-Acetyl 16*α*-hydroxytrametenolic acid, (2) dehydropachymic acid, (3) pachymic acid, (4) trametenolic acid, (5) dehydrotrametenolic acid.

Considering the hydrophobicity of triterpene acids, the effect of 40%, 60%, 80%, and 100% MeOH on the enrichment efficiency of the five triterpene acids was investigated. As shown in Figure 7, 80% MeOH led to loss of 3-O-acetyl-16*α*-hydroxytrametenolic acid and dehydropachymic acid, while 100% MeOH achieved high levels of recovery for all five triterpene acids. Thus, 60% and 100% MeOH were used for washing and elution steps, respectively.

### 3.6. Validation of the Phenylethanoid Glycosides and Triterpene Acids

For the analysis of triterpene acid and triterpene acid composition, the evaluated validation parameters including linearity, limits of detection (LODs), precision (repeatability), and recovery were examined (Table 2). The chromatographic method showed a favorable linearity in the concentration ranges assessed. The regression equation of purpureaside C was *Y* = 9.8161*X* + 0.8779 with a linear range of 2.24–56.00 *μ*g/mL, and the regression equation of jionoside A1 was *Y* = 8.9245*X* + 2.5501 with a linear range of 2.21–55.25 *μ*g/mL. The regression equation of acteoside was *Y* = 32.557*X* + 2.4327 with a linear range of 2.31–57.75 *μ*g/mL, and the regression equation of isoacteoside was *Y* = 13.012*X* − 4.3485 with a linear range of 1.16–29.00 *μ*g/mL. The regression equation of 3-O-actyl-16*α*-hydroxytrametenolic acid was *Y* = 6.6677*X* − 0.5066 with a linear range of 1.08–43.2 *μ*g/mL. The regression equation of dehydropachymic acid was *Y* = 6.2417*X* + 0.0102 with a linear range of 1.32–52.8 *μ*g/mL. The regression equation of pachymic acid was *Y* = 6.4675*X* − 1.3874 with a linear range of 4.41–165.6 *μ*g/mL. The regression equation of trametenolic acid was *Y* = 6.4439*X* − 0.4147 with a linear range of 3.78–151.2 *μ*g/mL. The regression equation of dehydrotrametenolic acid was *Y* = 6.0755*X* + 1.626 with a linear range of 2.7–108 *μ*g/mL.

The reproducibility of the experimental procedure was evaluated by carried out seven replicate samples during the same day. The RSDs of the experimental replicates were in the range of 0.35%–1.08%. The sensitivity of the method was verified by calculating 3 signal-to-noise ratios as LOD values and by calculating 10 signal-to noise ratios as LOQ values.

### 3.7. Analysis of LDC Samples

The method reported here was utilized for the simultaneous determination of the four phenylethanoid glycosides and five triterpene acids in LDC samples purchased from the market with different manufacturers (*n* = 18). As shown in Table 3, the average content of phenylethanoid glycosides and triterpene acids was 53.0 *μ*g/0.3 g and 115.9 *μ*g/0.3 g per capsule, respectively. The content of total triterpenoid acid is higher than that of total phenylethanolic glycoside in LDC; one of the reasons may be related to the manufacturing process of LDC that herbal *Poria* cocos is used as excipients after being crushed into powder, while other herbs are extracted by water and mixed with herbal *Poria* cocos powder (excipients) evenly to make capsules [5].

## 4. Conclusions

In this study, two HPLC-UV methods were established to determine the content of four phenylethanoid glycosides and five triterpene acids in LDC based on the novel use of SPE. The results showed that the LOD, LOQ, accuracy, and precision of the two methods met the requirements for quantitative analysis. A total of 18 batches of LDCs from six manufacturers were analyzed, demonstrating the utility of our methods for the routine analysis of formula quality and safety.

## Figures and Tables

**Table 1:** Recoveries of the four phenylethanoid glycosides and five triterpene acids from spiked samples using different SPE cartridges.

Compound	Recovery ± SD^*α*^ (%)				
	C18	Alumina-N	HLB	PSA	P-SAX
Purpureaside C	91.7 ± 0.5	19.5 ± 2.8	96.7 ± 2.2	64.0 ± 2.7	
Jionoside A1	92.2 ± 0.6	24.8 ± 4.1	97.3 ± 1.7	65.2 ± 2.3	
Acteoside	95.5 ± 1.2	12.4 ± 3.1	90.4 ± 0.4	88.6 ± 0.4	
Isoacteoside	90.3 ± 0.8	22.1 ± 3.3	93.6 ± 1.1	81.7 ± 1.8	
3-O-Acetyl 16*α*-hydroxytrametenolic acid	91.8 ± 0.2	15.6 ± 3.2	92.2 ± 2.5		62.0 ± 2.3
Dehydropachymic acid	96.6 ± 0.3	64.5 ± 2.4	96.8 ± 0.7		ND^b^
Pachymic acid	97.8 ± 1.5	71.3 ± 2.1	90.6 ± 1.7		70.3 ± 0.9
Trametenolic acid	95.2 ± 0.6	56.1 ± 2.1	93.5 ± 2.1		88.6 ± 0.8
Dehydrotrametenolic acid	94.5 ± 1.2	66.3 ± 2.2	90.2 ± 1.4		85.7 ± 1.5

^*α*^Standard deviation, *n* = 3. ^b^Below the detection limit.

**Table 2:** Method validation parameters of the four phenylethanoid glycosides and five triterpenes.

Compound	Intraday precision (RSD%), *n* = 6	Interday precision (RSD%), *n* = 3	Regression equation *Y* = *aX* + *b*^a^	Correlation coefficient (*r*)	Recovery ± SD^b^ (%)	LOD (*μ*g/mL)	LOQ (*μ*g/mL)
Purpureaside C	0.72	0.98	*Y* = 9.8161*X* + 0.8779	0.9999	93.7 ± 0.5	0.0044	0.0125
Jionoside A1	0.65	0.87	*Y* = 8.9245*X* + 2.5501	0.9998	94.2 ± 0.6	0.0031	0.0124
Acteoside	0.58	0.64	*Y* = 15.767*X* − 9.6178	0.9995	95.5 ± 1.2	0.0027	0.0081
Isoacteoside	0.82	1.02	*Y* = 13.012*X* − 4.3485	0.9996	93.3 ± 0.8	0.0028	0.0076
3-O-Acetyl-16*α*-hydroxytrametenolic acid	0.35	0.68	*Y* = 6.6677*X* − 0.5066	0.9998	91.8 ± 0.2	0.052	0.186
Dehydropachymic acid	0.62	1.05	*Y* = 6.2417*X* + 0.0102	0.9999	96.6 ± 0.3	0.073	0.205
Pachymic acid	0.84	1.08	*Y* = 6.4675*X* − 1.3874	0.9998	97.8 ± 1.5	0.136	0.418
Trametenolic acid	0.46	0.85	*Y* = 6.4439*X* − 0.4147	0.9996	95.2 ± 0.6	0.087	0.252
Dehydrotrametenolic acid	0.55	0.92	*Y* = 6.0755*X* + 1.626	0.9997	94.5 ± 1.2	0.064	0.198

^a^ *Y* is the peak area while *X* is the concentration (*μ*g/mL). ^b^Standard deviation, *n* = 3.

**Table 3:** Determination of the four phenylethanoid glycosides and five triterpene acids in 18 batches of LDC samples from six manufacturers (*n* = 3).

Sample	Content (*μ*g/capsule)										
	Purpureaside C	Jionoside A1	Acteoside	Isoacteoside	Total phenlyethanoid glycosides	3-O-Acetyl-16*α*-hydroxytrametenolic acid	Dehydropachymic acid	Pachymic acid	Trametenolic acid	Dehydrotrametenolic acid	Total triterpene acids
LDC01	10.8	14.3	18.2	8.1	51.4	3.0	13.0	82.6	13.2	4.0	115.8
LDC02	10.2	16.4	20.1	8.9	55.6	3.0	12.7	74.8	11.3	3.8	105.6
LDC03	12.0	15.3	19.5	8.6	55.4	3.2	12.2	57.2	14.4	3.6	90.6
LDC04	8.3	10.5	17.8	7.4	44.0	4.5	20.1	72.2	29	23.2	149
LDC05	8.8	11.4	18.3	7.5	46.0	4.1	20.7	65.0	22.3	20.9	133
LDC06	9.7	11.9	18.8	8.2	48.6	3.4	20.2	63.6	21.8	20.1	129.9
LDC07	13.2	15.6	23.4	10.1	62.3	1.8	27.7	75.8	16.4	11.3	133.0
LDC08	12.6	14.7	22.8	9.8	59.9	1.8	27.6	74.6	15.6	10.8	130.5
LDC09	14.1	16.9	24.5	9.7	65.2	1.9	27.7	74.7	16.9	11.8	132.9
LDC10	13.6	13.7	25.1	9.2	61.6	1.6	34.0	58.5	29.9	13.9	137.9
LDC11	12.8	13.1	24.8	9.4	60.1	1.6	33.4	58.1	28.7	13.7	135.4
LDC12	11.3	14.1	25.9	8.7	60.0	1.6	33.6	57.0	30.6	13.7	136.5
LDC13	10.7	13.7	20.5	8.5	53.4	1.4	11.2	44.6	6.1	5.5	68.7
LDC14	11.5	12.4	23.1	8.9	55.9	1.3	11.7	46.5	6.5	5.4	71.5
LDC15	9.7	10.1	19.3	7.8	46.9	1.4	10.8	45.1	6.0	5.9	69.3
LDC16	8.4	9.2	16.5	6.9	41.0	3.4	19.0	53.5	23.0	17.7	116.5
LDC17	7.9	8.6	16.9	6.2	39.6	3.0	18.6	50.1	22.1	19.8	113.6
LDC18	9.8	9.3	18.8	9.1	47.0	3.5	18.5	54.5	23.0	17.3	116.8

## Data Availability

The data used to support this study was obtained from the Shanghai University of Traditional Chinese Medicine, Shanghai, China, and are available from the corresponding author upon request.

## Abbreviations

HPLC-UV: High-performance liquid chromatography equipped with ultraviolet detector
SPE: Solid phase extraction
LD: Liuwei Dihuang preparation
LDC: Liuwei Dihuang capsule
